# The Superfund Basic Research Program—A Time of Change

**Published:** 2005-07

It is a time of new beginnings for the Superfund Basic Research Program, a university-based grants program established in 1987. While maintaining the program’s premise of supporting basic research for practical application to address the problems associated with the nation’s hazardous waste sites, the program continues to evolve and develop new approaches to address these concerns. An important component of achieving the program’s goals is the routine recompetition of the program. We are pleased to announce that as a result of the most recent recompetition, NIEHS has made awards to nine programs. Included in these nine awards are one new grantee, Brown University, and eight grantees that are continuing participants in the program. These grantees include Boston University, Dartmouth College, Duke University, Texas A & M University, the University of Arizona, the University of California at Davis, the University of California at San Diego, and the University of Kentucky. These nine awards in addition to the eleven existing grantees comprise the current Superfund Basic Research Program.

The new program has a strong emphasis on interdisciplinary and system approaches to addressing the complex issues associated with hazardous waste sites. Grantees are using integrated research models to understand how contaminants are transformed as they move through soils, sediments, and groundwater and how they interact with ecosystems and ultimately affect human health. These robust research efforts are augmented with graduate and postdoctoral training in interdisciplinary environmental research and with community outreach activities. Each grantee is also required to engage proactively in research translation to other federal agencies, industry, and the community at large.

For interested investigators who are not already part of this vital and exciting program but would like to be, please contact program staff about future opportunities. We anticipate that there will be annual solicitations beginning in the summer of 2006. It is not too early to start the planning process! We are available to assist you as you begin to conceptualize a future program at your university.

## Contact

**William Suk, PhD, Director** | (919) 541-0797

**Claudia Thompson, PhD** | (919) 541-4638

**Beth Anderson** | (919) 541-4481

## Figures and Tables

**Figure:**
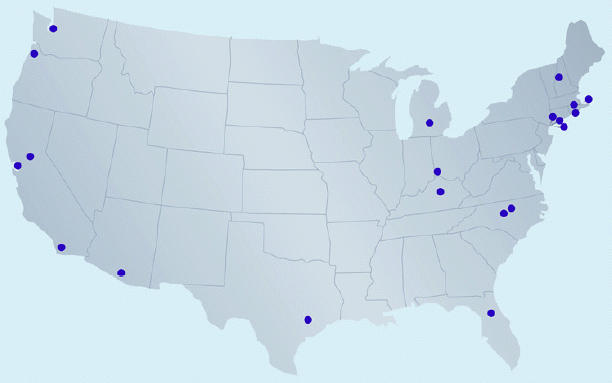
Superfund Program sites.

**Figure:**
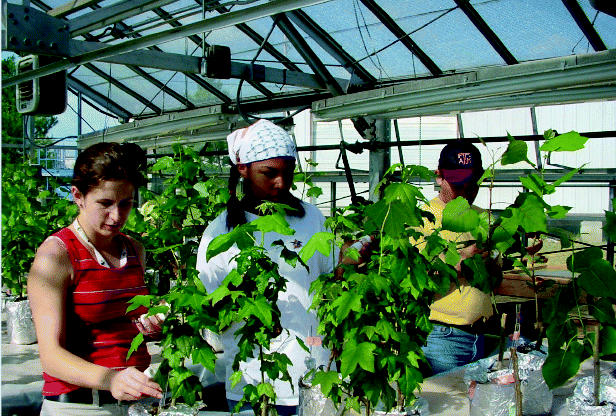
Students participating in a phytoremediation project as part of the University of Washington program.

